# Sorting Center Value Identification of “Internet + Recycling” Based on Transfer Clustering

**DOI:** 10.3390/s22197629

**Published:** 2022-10-08

**Authors:** Cheng Cheng, Xiaoli Luan

**Affiliations:** Key Laboratory of Advanced Process Control for Light Industry, Jiangnan University, Wuxi 214122, China

**Keywords:** value identification, transfer clustering, inter-class balanced data selection, *GFMR* model, Internet + Recycling

## Abstract

As the core link of the “Internet + Recycling” process, the value identification of the sorting center is a great challenge due to its small and imbalanced data set. This paper utilizes transfer fuzzy c-means to improve the value assessment accuracy of the sorting center by transferring the knowledge of customers clustering. To ensure the transfer effect, an inter-class balanced data selection method is proposed to select a balanced and more qualified subset of the source domain. Furthermore, an improved *RFM* (Recency, Frequency, and Monetary) model, named *GFMR* (Gap, Frequency, Monetary, and Repeat), has been presented to attain a more reasonable attribute description for sorting centers and consumers. The application in the field of electronic waste recycling shows the effectiveness and advantages of the proposed method.

## 1. Introduction

In 2015, China’s recycling pattern shifted from “manual recycling” to “Internet + Recycling” [1]. The “Internet + Recycling” process is very important to identify the value of the sorting center [2]. Take electronic waste (e-waste) recycling as an example. The company will offer information to sorting centers and receive a commission [3]. Therefore, the “Internet + Recycling” companies will design a specific strategy based on sorting center costs [4], recycling channels [5], and value dimensions [6]. In this way, the company can improve its business competitiveness and reduce churn at the sorting center. However, the data set of sorting center is small and imbalanced that is called “Absolute Rarity”, [7] which makes it difficult to carry out the task of value assessment. The traditional oversampling methods [8] may not evaluate the accurate value for sorting centers.

Transfer learning is a branch of machine learning that has been shown to help solve problems with small data sets. It has been widely used in image classification [9], signal processing [10], and text classification [11]. An effective model for the target domain can be obtained by leveraging useful relative information from the source domain. However, there is still limited research on the transfer clustering problem. Jiang et al. proposed transfer spectral clustering (TSC), which could transfer knowledge from related clustering task [12]. Wang et al. extended three traditional Gaussian mixture model (GMM) to transfer clustering versions [13]. These methods are more suitable for the clustering problem that has definite boundaries. However, the amount of sorting centers is too limited to obtain boundaries. Fuzzy c-means (FCM) [14] is a clustering algorithm that could solve this problem. The algorithm are more applicable in many fields by changing the objective function of the FCM [15]. Transfer fuzzy c-means (TFCM) [16] is a transfer clustering version of FCM which has good performance on small data set clustering by transferring knowledge from the relative source domain’s cluster centers. There are plentiful customers in “Internet + Recycling” which contain useful and relative information. Thus, in this paper, we adopt TFCM to transfer the cluster centers of customers as knowledge to assist cluster sorting centers. In order to achieve accurate cluster centers, a comprehensive model that can describe the characteristics of customers is necessary. The RFM model with clustering algorithm has been widely used in customer value identification. Pondel et al. compared three different clustering algorithms’ results of 56,237 customers who made at least 2 purchases in the online store [17]. Kumar et al. practiced the RFM model on 127,037 business customer [18]. Many other scholars also adjust the RFM model according to the features of customers. For example, Li et al. used an improved RFM model with added indicators to classify 4000 customers on the e-commerce platform [19]. Fahed et al. used an enhanced RFM model to classify 42,172 retail customers [20]. However, in the “Internet + Recycling” process, some high-value customers are the individual economy which is rare in customers. The original RFM model could not describe a comprehensive characterization of the high-value customers.

In summary, to get the accurate value identification of sorting centers, TFCM that transferring knowledge from customers is adopted to solve the problem of the small amount of data set, and inter-class balanced data selection (IBDS) is proposed to help solve the problem of imbalanced data set. In order to obtain accurate customer cluster centers for transfer, an improved RFM model GFMR is proposed. The application in “Internet + Recycling” company proves our approach can effectively improve the accuracy of classifying sorting centers. With the accurate value identification of sorting centers, “Internet + Recycling” companies can apply their marketing strategies more precisely, which could improve business competitiveness and reduce sorting center churn.

## 2. Acquisition of Customer Cluster Centers

Accurate cluster centers of customers is the prerequisite for TFCM. The *RFM* model is a popular customer value analysis tool widely used to measure customer lifetime value as well as customer value identification and behavioral analysis. The original RFM definition is as follows:

*R*: recency of the last trade
*F*: frequency of trades
*M*: monetary value of the trades

However, the original RFM model could not identify the active customer. The *R* of RFM is almost the same for a new customer and an active loyal customer. In the “Internet + Recycling” company, the top 20% customers who are active in trade devote over 60% trading volume. Therefore, it is important to divide high-value customers from others.

The high-value customers of “Internet + Recycling” companies are sometimes the individual economy. They will recycle some specific goods and store them. When the price of the goods is relatively high, they will place several orders online. Therefore, their characteristics are short trade gap, high frequency, huge monetary value, and focus on particular goods. In order to strengthen the ability to identify high-value customers, this paper proposed GFMR model as follows.

*G*: average trade gap time between two trade
*F*: frequency of trade
*M*: monetary value of all trade
*R*: maximum number of repeat transactions for the same goods

Especially, *G* is defined as in Equation (1)

(1) $$G=\left\{\begin{array}{cc} \;\;\;T_{s} & \;\mathrm{if}\;F=1\\ \frac{T_{f}-T_{l}}{F} & \;\mathrm{if}\;F\neq 1 \end{array}\right.$$

where $T_{s}$ denotes the statistical interval, $T_{f}$ denotes the first date the consumer traded during the statistical interval, $T_{l}$ denotes the last date the consumer traded during the statistical interval. If a customer has only traded one time during the $T_{s}$, this paper assumes the consumer’s average trade gap is bigger than the statistical interval set $G=T_{s}$. Otherwise, calculate the true trade gap.

The GFMR model will reduce the effect of randomness because the four indicators all have small relation to the sampling date. The average gap will separate intensive trade consumers from the others. The frequency and monetary value will identify loyal consumers. The repeat recycling times could identify individual economies. Consequently, GFMR is more suitable for identifying the consumer value of “Internet + Recycling”. Based on GFMR model, k-means algorithm [21] is used to obtain the cluster centers.

Definition of variables:

$D_{S}=\left\{x_{j}^{S}\right\}$(j=1,2,…,M): domain consisting of the standardized GFMR data of *m* customers

*K*: the number of clusters

${\tilde{v}}_{k}$: the *k*th cluster center of customers

Steps of acquiring customer cluster centers by k-means algorithm are as followed:

Step1: Randomly generate ${\tilde{v}}_{k}$(k=1,2,…,K) as initial cluster centers.

Step2: Calculate the distance of each $x_{j}^{S}$ to ${\tilde{v}}_{k}$ as ${∥x_{j}^{S}-{\tilde{v}}_{k}∥}^{2}$ and classify the sample into the cluster corresponding to the minimal distance.

Step3: Calculate the mean value of all samples within each cluster and update the ${\tilde{v}}_{k}$.

Step4: Repeat step2 and step3 until the maximum number of iterations.

The final $V=\{{\tilde{v}}_{1},{\tilde{v}}_{2},\ldots ,{\tilde{v}}_{K}\}$ is the customer cluster centers.

## 3. Transfer Clustering for Sorting Centers

The data set of the introduced customer clustering study ranged from 4000 to 127,037, the amount of sorting centers is only 223 which is far from the modeling order of magnitude. In transfer learning, the domain containing a large amount of useful information is often defined as the source domain. And the domain we need to learn is defined as the target domain. In this paper, the customers data set is the source domain $D_{S}=\left\{x_{j}^{S}\right\}\left(j=1,2,\ldots ,M\right)$ and the labeled sorting centers data set is the target domain $D_{T}=\left\{(x_{i}^{T},L\left(x_{i}^{T}\right))\right\}\left(i=1,2,\ldots ,N\right)$.

Due to the small size of the data set, there are no legible boundaries between each class. FCM could help solve this problem. The objective function of the original FCM is as follows.

(2) $$\begin{array}{c} \min_{U,V}\;J_{FCM}=\sum_{k=1}^{K}\sum_{i=1}^{N}u_{ik}^{\alpha }{∥x_{i}^{T}-v_{k}∥}^{2}\\ st.u_{ik}\in \left[0,\;1\right],\;\sum_{k=1}^{K}u_{ik}=1,\;0<\sum_{i=1}^{N}u_{ik}<N \end{array}$$

where *K* denotes the number of clusters, $U={\left[u_{ik}\right]}_{K\times N}$ is the fuzzy/possibilistic partition matrix whose element $u_{ik}$ denotes the membership of the *i*th sample belonging to the *k*th class, α denotes the fuzzy index, $V={\left[v_{1},v_{2},\ldots ,v_{K}\right]}^{T}$ is the matrix of K cluster centers whose element $v_{k}$ denotes the *k*th cluster center of sorting centers.

Transferring knowledge from the $D_{S}$ is a must because the $D_{T}$ cannot be trained to a satisfactory model on its own. There is a transfer learning version of FCM in which the objective function is defined as followed [16].

(3) $$\begin{array}{c} \min_{U,V}\;J_{TFCM}=\sum_{k=1}^{K}\sum_{i=1}^{N}u_{ik}^{\alpha }{∥x_{i}^{T}-v_{k}∥}^{2}+\\ \lambda_{1}\cdot \sum_{k=1}^{K}\sum_{i=1}^{N}u_{ik}^{\alpha }{∥x_{i}^{T}-{\tilde{v}}_{k}∥}^{2}+\lambda_{2}\cdot \sum_{k=1}^{K}\left(\sum_{i=1}^{N}u_{ik}^{\alpha }\right){∥{\tilde{v}}_{k}-v_{k}∥}^{2}\\ st.u_{ik}\in \left[0,\;1\right],\;\sum_{i=k}^{K}u_{ik}=1,\;0<\sum_{i=1}^{N}u_{ik}<N \end{array}$$

where $\lambda_{1}$ and $\lambda_{2}$ are non-negative balance parameters.

The learning rules based on Equation (3) are as follows:

(4) $$v_{k}=\frac{\sum_{i=1}^{N}u_{ik}^{\alpha }x_{i}+\lambda_{1}\sum_{i=1}^{N}u_{ik}^{\alpha }\tilde{v_{k}}}{\sum_{i=1}^{N}u_{ik}^{\alpha }+\lambda_{2}\sum_{i=1}^{N}u_{ik}^{\alpha }}$$

(5) $$u_{ik}\;=\;\frac{\left(\frac{1}{{∥x_{i}^{T}\;-\;v_{k}∥}^{2}\;+\;\lambda_{1}{∥x_{i}^{T}\;-\;{\tilde{v}}_{k}∥}^{2}\;+\;\lambda_{2}{∥\tilde{v_{k}}\;-\;v_{k}∥}^{2}}\right){\;}^{\frac{1}{m-1}}}{\sum_{k=1}^{C}\;\left[\frac{1}{{∥x_{i}^{T}\;-\;v_{k}∥}^{2}\;+\;\lambda_{1}{∥x_{i}^{T}\;-\;{\tilde{v}}_{k}∥}^{2}\;+\;\lambda_{2}{∥\tilde{v_{k}}\;-\;v_{k}∥}^{2}}\right]{\;}^{\frac{1}{m-1}}}$$

There TFCM Algorithm 1 is described below.
**Algorithm 1:** TFCM   1. Initialize the number of iterations as *t* = 0 and the $u_{ik}$ randomly; Set the maximum number of iterations $t_{max}$ and threshold ϵ; Set the balance parameters $\lambda_{1}$ and $\lambda_{2}$;   2. Update the $v_{k}\left(t\right)$ using Equation (4)   3. Set t=t+1;   4. Update the $u_{ik}\left(t\right)$ using Equation (5)   5. If all $∥u_{ik}\left(t\right)-u_{ik}\left(t-1\right)∥<\epsilon$ or $t=t_{max}$, then terminate; else go to 2.

The computational complexities of TFCM is O(tNK+tC) that is the same as FCM.

This method transfers knowledge from the source domain to the target domain through source domain cluster centers. Changing $\lambda_{1}$ and $\lambda_{2}$ could adjust the level of learning from the source domain. As is proved in the article [16], if the source domain has bad knowledge, it will have a negative influence on the clustering performance in the target domain, which is called negative transfer. The original article tried and failed to adopt appropriate parameter values for reducing the effect of the bad source domain. Therefore, in this paper we choose data selection methods rather than adopting parameter values that have been proven to be effective.

In data selection, the key is to find a measurement between the source and target domain. Kullback–Leibler (KL) divergence is often used to measure the difference between two distributions. The KL divergence is defined as follows.

(6) $$\begin{array}{c} D_{KL}\left(p||q\right)=\sum_{i=1}^{}p\left(x_{i}\right)\log_{}\frac{p(x_{i})}{q(x_{i})}\\ \;\;\;=\sum_{i=1}^{}p\left(x_{i}\right)\log_{}p(x_{i})-\sum_{i=1}^{}p\left(x_{i}\right)\log_{}q(x_{i})\\ \;\;\;=H(p,q)-H(p) \end{array}$$

where H(p,q) denotes the cross-entropy, H(p) denotes the information entropy.

In $D_{KL}\left(p||q\right)$, the $H_{p}$ is constant. And proved by Gibbs’ inequality that H(p,q) is bigger than $H_{p}$, so $D_{KL}\left(p||q\right)$ is monotonic and H(p,q) could represent $D_{KL}\left(p||q\right)$. The smaller H(p,q) means *q* is closer to *p*.

In this paper, assume the distribution of the source domain is *p* which has *m* samples, and the distribution of the target domain is *q* which has *n* samples. In this paper, we attempt to find source domain samples that are more similar to the target domain. Turn into the math equation, the smaller H(q,p) is what we want. So order the source domain by $H\left(q,p,i\right)=-q\left(x_{i}\right)\log_{}p(x_{i})$, and select the relative smaller *s* samples to compose the subset of source domain. It is easy to obtain:

(7) $$H^{{}^{\prime }}\left(p,q\right)=-\sum_{i=1}^{s}q\left(x_{i}\right)\log_{}p(x_{i})<H\left(p,q\right)$$

which means we can measure source domain samples by H(q,p,i), the smaller the sample is closer to the target domain.

**Figure 1:**
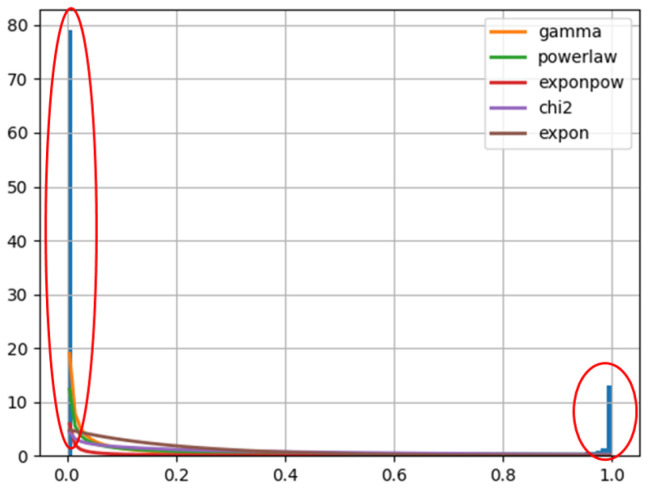
Fitted Distributions of G of Customers.

However, due to the imbalanced number of different categories, if not separate the target domain or the source domain and calculate the distribution individually, a class with a larger amount will overwrite the features of a class with a smaller amount. As is shown in Figure 1, when fitting the distribution of G of consumers. The five best-fitting distributions all neglect small samples.

This paper proposes an inter-class balanced data selection method(IBDS). The steps of IBDS are given in Algorithm 2.
**Algorithm 2:** IBDS**Input:** labeled target domain $D_{T}$, unlabeled source domain $D_{S}$   1. Separate $D_{T}$ by $L(x_{i}^{T})$ for $\left\{D_{T}^{k}\right\}$(k=1,2,…,K)   2. Calculate the geometric center of $\left\{D_{T}^{k}\right\}$ for $C_{T}=\left\{c_{k}^{T}\right\}$   3. Calculate the distance between $x_{j}^{S}$ and $C_{T}$ for $Dis_{j}=\left\{{\mathrm{dis}}_{j}^{k}\right\}$   4. Classify $x_{j}^{S}$ by minimum $Dis_{j}$ to $\left\{D_{S}^{k}\right\}$   5. Fit distribution of $\left\{D_{T}^{k}\right\}$ for $\left\{P_{T}^{k}\right\}$   6. Fit distribution of $\left\{D_{S}^{k}\right\}$ for $\left\{Q_{S}^{k}\right\}$   7. In k class calculate the H(q,p,i)   8. Order $\left\{D_{S}^{k}\right\}$ by H(q,p,i) the smaller the better   9. Taking the smallest sample size in each category as *s*   10. Combine the first *s* samples of each category for $D_{S}^{{}^{\prime }}$**Output:**$D_{S}^{{}^{\prime }}$

The computational complexities of IBDS is $O(K^{2}M+bN)$, where *b* is the number of features of $D_{T}$. The proposed algorithm will increase the complexity of the algorithm to some extent, but it can significantly improve the overall accuracy of the algorithm.

This paper ordered the source domain samples by calculating H(q,p,i) for each sample in a different category. Then, use the best *s* samples of each category to get a balanced and more similar subset that provides better cluster center TFCM. The method has proved to be effective.

## 4. Experimental Results

In this section, the proposed algorithm is evaluated on real-world data set. This paper collected 754,904 e-waste recycling order records of consumers and 19,703 e-waste transporting records of sorting centers from January 2021 to December 2021 of China’s “Internet + Recycling” company. 308,059 consumers and 223 sorting centers were detected. The company has developed four marketing programs targeting high-value, potential-value, stable-value and low-value sorting centers. The goal of this paper is to accurately identify 4 types of sorting center values.

### 4.1. Data Processing

The consumer is modeled by GFMR. Due to the trade of sorting centers, it is always counted by cars without the specific trade category. So, the model used is the GFM model. The data is normalized by

(8) $$x^{{}^{\prime }}=\frac{x_{max}-x}{x_{max}-x_{min}}$$

(9) $$x^{{}^{\prime }}=\frac{x-x_{min}}{x_{max}-x_{min}}$$

The smaller *G* is better. Thus *G* is normalized by Equation (8). *F*, *M*, and *R* are the bigger the batter, so they are normalized by Equation (9). $G^{{}^{\prime }}$, $F^{{}^{\prime }}$, $M^{{}^{\prime }}$, $R^{{}^{\prime }}$ are the standardized variables.

### 4.2. Customer Value Identification

Based on the RFM and GFMR model, results of the k-means cluster algorithm to identify the value of customer are as follows.

As is shown in Table 1, the difference of *R* between four clusters is not obvious. Thus, the algorithm may incorrectly classify some high-value users into potential-value user groups, which leads to the high *M* of potential-value. Between stable-value and low-value, the difference between these two clusters is mainly the *R* which is caused by the random time the new customer begins to use the service. As is shown in Table 2, the difference of *G* between four clusters is very obvious, and the added *R* vivid segments high-value customers from the others. Low-value customers who trade only once are clearly separated from stable-value users who trade more actively. Thus, the result of the GFMR model is better than the RFM model. The cluster centers are more informative for sorting centers clustering.

### 4.3. Sorting Center Value Identification

However, there is still a large gap between clustering centers of the source domain and target domain, as shown in Table 3 and Table 4.

This means that there is a great difference between the source domain and the target domain. Therefore, IBDS is practiced to find a more appropriate source domain. The cluster centers of $D_{S}^{{}^{\prime }}$ are shown in Table 5.

**Figure 2:**
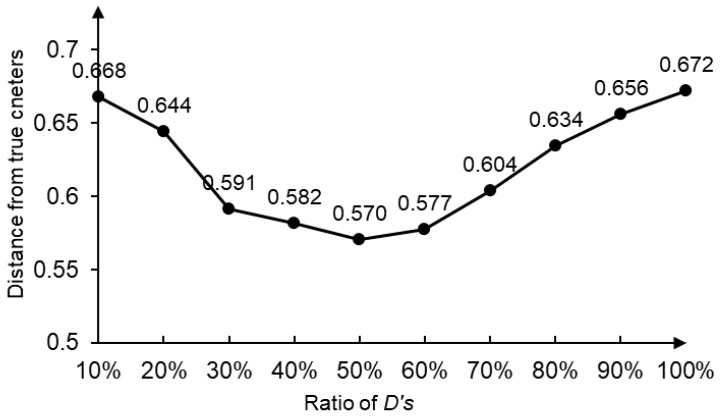
Variation of distance between true centers and clustering centers.

As is shown in Table 5, the four clustering centers are all closer to the target domain. IBDS orders samples by H(q,p,i), the smaller, the more similar to the target domain. The variation of distance between the four clustering centers and the true target domain centers with different top ratios of $D_{S}^{{}^{\prime }}$ is shown in Figure 2.

As is shown in Figure 2, when taking 50% of $D_{S}^{{}^{\prime }}$ the distance is minimum. If the ratio is too large, the subset will contain some samples that are not very similar to the target domain. If the ratio is too small, the randomness of the samples will also affect the similarity between the subset and the target domain.

To prove the effect of IBDS, this paper also presents the accuracy of transferring the knowledge of $D_{S}$ and 50% of $D_{S}^{{}^{\prime }}$ with $t_{max}=100$ in Table 6 and Table 7. A greener background color in Table 6 and Table 7 means higher accuracy, and a redder background color means lower accuracy.

As is shown in Table 6, compared to 100% of $D_{S}^{{}^{\prime }}$, the accuracy of top 50% of $D_{S}^{{}^{\prime }}$ with the same $\lambda_{1}=10$ and $\lambda_{2}=1$ is 95.07% which is higher than 91.03%. Compared to the original $D_{S}$, the results of $D_{S}^{{}^{\prime }}$ have higher accuracy in most parameters. $D_{S}^{{}^{\prime }}$ exceed $D_{S}$ 7.13% of all kinds of parameters. When $\lambda_{1}$ is bigger than $\lambda_{2}$, it usually has good accuracy because $v_{k}$ is effected by the randomness of small data set. This impact can be reduced by enhancing the learning of high quality data in the source domain. Thus, IBDS combined with TFCM effectively clusters small and imbalanced data sets.

**Figure 3:**
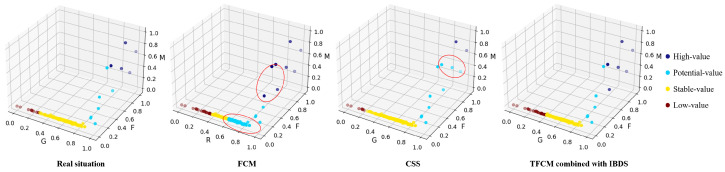
Comparison of different clustering approaches.

To highlight the advantages of our approach, we also compared it with FCM and CSS (clustering with stratified sampling technique) [8]. The detailed results are shown in Figure 3.

Obviously, TFCM combined with IBDS could get the result that is most close to real situation. The data set of the sorting center is small and imbalanced. Thus, the accuracy of FCM is only 60.09%. FCM classifies some high-value sorting centers into the potential-value sorting centers and classifies some potential-value sorting centers into the stable-value sorting center because setting the cluster centers in the dense samples could achieve a lower score of the Equation (2). CSS is an imbalanced data classification algorithm. The accuracy of CSS is 83.41%. CSS is more accurate in identifying low-value sorting centers. But TFCM combined with IBDS outperforms CSS in identifying high-value sorting centers. Because the small data set is easily overfitted by the way of generating samples through oversampling. Transfer learning can effectively improve the accuracy of sorting center value identification and avoid overfitting at the same time.

## 5. Conclusions

Considering the fact that the data set of sorting centers is small and imbalanced, TFCM combined with IBDS has been proposed to solve the value identification problem. TFCM could transfer knowledge from clustering centers of customers. The IBDS could find a subset that are more similar and balanced than the target domain. In further research, different ratios of the subset exhibits different disparities from the target domain which is caused by randomness and redundant samples. A suitable ratio that balances sample diversity and validity will have better performance. In most of the parameters, IBDS elevates the accuracy which proved the validity of the method. Compared with FCM, the value assessment accuracy of the sorting center elevated from 60.09% to 91.03%. The method in this paper is also less likely to be overfitted compared to the oversampling method.

Further research will focus on automatic adjustment of the ratio to balance sample diversity and validity. In this paper, customers and sorting centers share similar characteristics. However, transferring knowledge from data sets without similar features is still a challenge.

## Figures and Tables

**Table 1:** Clustering customer by RFM.

Cluster	Count	*R*	*F*	*M*
High-value	165	119.3455	304.3000	16,427.7000
Potential-value	1146	124.1798	90.7749	5467.7400
Stable-value	153,094	95.2453	1.7242	96.5492
Low-value	153,654	286.7248	1.4013	76.7217

**Table 2:** Clustering customer by GFMR.

Cluster	Count	*G*	*F*	*M*	*R*
High-value	543	0.9715	193.9931	9243.0920	156.0792
Potential-value	57,683	5.5829	4.6739	263.0061	4.1112
Stable-value	6337	76.1596	2.5564	193.2928	1.7175
Low-value	243,496	364.9982	1.0000	56.2338	1.0000

**Table 3:** True centers of target domain.

Cluster	$G^{{}^{\prime }}$	$F^{{}^{\prime }}$	$M^{{}^{\prime }}$
High-value	0.9967	0.7258	0.7035
Potential-value	0.9718	0.1771	0.3338
Stable-value	0.5945	0.0073	0.0107
Low-value	0.1865	0.0036	0.0059

**Table 4:** Cluster centers of $D_{S}$.

Cluster	$G^{{}^{\prime }}$	$F^{{}^{\prime }}$	$M^{{}^{\prime }}$	Distance
High-value	0.9922	0.3373	0.2806	0.5742
Potential-value	0.9847	0.0070	0.0067	0.3689
Stable-value	0.7912	0.0030	0.0041	0.1969
Low-value	0.0000	0.0000	0.0013	0.1866

**Table 5:** Cluster centers of $D_{S}^{{}^{\prime }}$.

Cluster	$G^{{}^{\prime }}$	$F^{{}^{\prime }}$	$M^{{}^{\prime }}$	Distance
High-value	0.9987	0.6216	0.4156	0.3062
Potential-value	0.9779	0.1483	0.2847	0.0572
Stable-value	0.6937	0.0021	0.0031	0.0997
Low-value	0.1392	0.0016	0.0035	0.0473

**Table 6:** Accuracy of transferring top 50% of $D_{S}^{{}^{\prime }}$ by TFCM.

	*λ*_2_	0	0.005	0.1	0.5	0.7	1	1.5	10	50	100	Average	Max
*λ*_1_													
0		30.04%	31.84%	25.56%	83.41%	77.13%	90.13%	3.59%	2.24%	2.24%	2.24%	34.84%	90.13%
0.005		29.15%	24.66%	25.56%	83.41%	77.13%	89.24%	3.59%	2.24%	2.24%	2.24%	33.95%	89.24%
0.1		69.51%	69.06%	2.24%	78.92%	75.78%	2.24%	3.59%	2.24%	2.24%	2.24%	30.81%	78.92%
0.5		82.96%	82.51%	89.69%	34.53%	52.47%	2.24%	3.59%	2.24%	2.24%	2.24%	35.47%	89.69%
0.7		86.10%	86.10%	91.93%	19.28%	28.25%	48.43%	2.24%	2.24%	2.24%	2.24%	36.91%	91.93%
1		88.79%	88.79%	92.83%	13.00%	16.14%	25.56%	2.24%	2.24%	2.24%	2.24%	33.41%	92.83%
1.5		91.48%	91.48%	92.38%	90.58%	10.31%	13.45%	3.59%	2.24%	2.24%	2.24%	40.00%	92.38%
10		96.41%	96.41%	96.41%	95.96%	95.07%	95.07%	94.17%	2.24%	2.24%	2.24%	67.62%	96.41%
50		96.41%	96.41%	96.41%	96.41%	96.41%	96.41%	95.96%	96.41%	2.24%	2.24%	77.53%	96.41%
100		96.41%	96.41%	96.41%	96.41%	96.41%	96.41%	96.41%	96.41%	97.31%	2.24%	87.09%	97.31%
Average		76.73%	76.37%	70.94%	69.19%	62.51%	55.92%	30.90%	21.08%	11.75%	2.24%	47.76%	

**Table 7:** Accuracy of transferring $D_{S}$ by TFCM.

	*λ*_2_	0	0.005	0.1	0.5	0.7	1	1.5	10	50	100	Average	Max
*λ*_1_													
0		30.04%	29.60%	38.57%	19.28%	11.21%	5.38%	3.59%	2.24%	2.24%	2.24%	14.44%	38.57%
0.005		28.70%	33.18%	37.67%	19.28%	11.21%	5.38%	3.59%	2.24%	2.24%	2.24%	14.57%	37.67%
0.1		44.84%	43.95%	18.39%	23.32%	13.45%	5.38%	3.59%	2.24%	2.24%	2.24%	15.96%	44.84%
0.5		78.92%	79.37%	71.75%	39.01%	26.91%	2.24%	2.24%	3.59%	2.24%	2.24%	30.85%	79.37%
0.7		82.51%	82.51%	78.92%	45.29%	31.39%	2.24%	2.24%	2.24%	2.24%	2.24%	33.18%	82.51%
1		84.75%	85.20%	89.69%	56.95%	41.26%	18.83%	4.48%	2.24%	2.24%	2.24%	38.79%	89.69%
1.5		87.89%	88.34%	91.48%	74.44%	56.05%	31.84%	7.17%	2.24%	2.24%	2.24%	44.39%	91.48%
10		92.83%	92.83%	91.93%	92.38%	92.38%	91.93%	91.03%	3.59%	2.24%	2.24%	65.34%	92.83%
50		92.83%	92.83%	92.83%	92.38%	92.38%	92.38%	92.38%	88.34%	3.59%	2.24%	74.22%	92.83%
100		92.83%	92.83%	92.83%	92.38%	91.93%	91.93%	91.93%	91.48%	3.59%	3.59%	74.53%	92.83%
Average		71.61%	72.06%	70.40%	55.47%	46.82%	34.75%	30.22%	20.04%	2.51%	2.38%	40.63%	

## Data Availability

The data presented in this study are available on request from the corresponding author.

## References

[1] Wang H., Han H., Liu T., Tian X., Xu M., Wu Y., Gu Y., Liu Y., Zuo T. (2018). “Internet+” recyclable resources: A new recycling mode in China. Resour. Conserv. Recycl..

[2] He K., Li L., Ding W. Research on recovery logistics network of waste electronic and electrical equipment in China. Proceedings of the 2008 3rd IEEE Conference on Industrial Electronics and Application.

[3] Liu T., Zhang Q., Zheng Z., Wu S., Weng Z. (2022). Stakeholder Analysis of the Waste Electrical and Electronic Equipment Internet Recycling Industry. Int. J. Environ. Res. Public Health.

[4] Jian H., Xu M., Zhou L. (2019). Collaborative collection effort strategies based on the “Internet+ recycling” business model. J. Clean. Prod..

[5] Qu Y., Zhang Y., Guo L., Cao Y., Zhu P. (2022). Decision Strategies for the WEEE Reverse Supply Chain under the “Internet+ Recycling” Model. Comput. Ind. Eng..

[6] Cui Y., Cao Y., Ji Y., Chang I.S., Wu J. (2022). Determinant factors and business strategy in a sustainable business model: An explorative analysis for the promotion of solid waste recycling technologies. Bus. Strategy Environ..

[7] Al-Stouhi S., Reddy C.K. (2016). Transfer learning for class imbalance problems with inadequate data. Knowl. Inf. Syst..

[8] Cao L., Shen H. (2022). CSS: Handling imbalanced data by improved clustering with stratified sampling. Concurr. Comput. Pract. Exp..

[9] Ju J., Zheng H., Xu X., Guo Z., Zheng Z., Lin M. (2022). Classification of jujube defects in small data sets based on transfer learning. Neural Comput. Appl..

[10] George D., Shen H., Huerta E. (2018). Classification and unsupervised clustering of LIGO data with Deep Transfer Learning. Phys. Rev. D.

[11] Liu Z.-G., Li X.-Y., Qiao L.-M., Durrani D.K. (2020). A cross-region transfer learning method for classification of community service cases with small datasets. Knowl.-Based Syst..

[12] Jiang W., Liu W., Chung F.l. (2018). Knowledge transfer for spectral clustering. Pattern Recognit..

[13] Wang R., Zhou J., Jiang H., Han S., Wang L., Wang D., Chen Y. (2021). A general transfer learning-based Gaussian mixture model for clustering. Int. J. Fuzzy Syst..

[14] Bezdek J.C., Ehrlich R., Full W. (1984). FCM: The fuzzy c-means clustering algorithm. Comput. Geosci..

[15] Pehlivan N.Y., Turksen I.B. (2021). A novel multiplicative fuzzy regression function with a multiplicative fuzzy clustering algorithm. Rom. J. Inf. Sci. Technol..

[16] Deng Z., Jiang Y., Chung F.L., Ishibuchi H., Choi K.S., Wang S. (2015). Transfer prototype-based fuzzy clustering. IEEE Trans. Fuzzy Syst..

[17] Pondel M., Korczak J. Collective clustering of marketing data-recommendation system Upsaily. Proceedings of the 2018 Federated Conference on Computer Science and Information Systems (FedCSIS).

[18] Kumar S.J., Philip A.O. Achieving Market Segmentation from B2B Insurance Client Data Using RFM & K-Means Algorithm. Proceedings of the 2022 IEEE International Conference on Signal Processing, Informatics, Communication and Energy Systems (SPICES).

[19] Li X., Li C. The research on customer classification of B2C platform based on k-means algorithm. Proceedings of the 2018 IEEE 3rd Advanced Information Technology, Electronic and Automation Control Conference (IAEAC).

[20] Yoseph F., Heikkila M. Segmenting retail customers with an enhanced RFM and a hybrid regression/clustering method. Proceedings of the 2018 International Conference on Machine Learning and Data Engineering (iCMLDE).

[21] Borlea I.D., Precup R.E., Borlea A.B. (2022). Improvement of K-means Cluster Quality by Post Processing Resulted Clusters. Procedia Comput. Sci..

